# Switched-Delay Smith Predictor for the Control of Plants with Response-Delay Asymmetry

**DOI:** 10.3390/s23010258

**Published:** 2022-12-27

**Authors:** Algirdas Baskys

**Affiliations:** Faculty of Electronics, Vilnius Gediminas Technical University, Sauletekio Av. 11, 10223 Vilnius, Lithuania; algirdas.baskys@vilniustech.lt

**Keywords:** nonlinear control systems, asymmetric dynamics, response delay, Smith predictor, frequency converters, water-supply systems

## Abstract

The modification of a Smith predictor for the control of plants with response-delay asymmetry has been proposed. It was developed for application in frequency converters for the control of the speed of the AC induction motor drives of pumps used in water- and liquefied-petroleum-gas-supply systems. Such plants are characterized by long response delays, and often these delays are asymmetric, i.e., the response delay to the rising and falling plant-control signal is different. A distinctive feature of the proposed modification is that the value of the response delay in the model of the plant used for the realization of the Smith predictor is switched. The operation of the proposed switched-delay Smith predictor, which was used with the proportional-integral controller, was analyzed using a simulation and experimentally in a real water-supply system. The obtained results prove an advantage of the suggested solution.

## 1. Introduction

In most feedback-control applications, the controlled plants are considered as objects with symmetric dynamics, i.e., considering that the dynamics of a plant are the same during the rise and fall of the plant’s output parameter. However, in practice, many actual plants are characterized by asymmetric dynamics. Generally, the dynamics of plants, in which the controlled parameter is increased by one mechanism and decreased by another, are asymmetric. For example, the speed of a vehicle is increased by the energy produced by the engine but decreased due to friction, air resistance or brakes; the temperature of a heated room is increased by the energy provided to a heater, but decreased by heat losses; the liquid pressure in a liquid-supply system is increased by the pump, but decreased because of the consumption of the liquid. The dynamics’ asymmetry causes a change in the parameters of the plant-transfer function or a change of the transfer function itself. If the dynamics’ asymmetry is significant, the use of controllers developed for the symmetric dynamics does not allow for us to achieve good control quality. 

There is not much literature devoted to the control systems of plants with asymmetric dynamics. References [1,2,3,4,5] can be mentioned. The literature [1] deals with plants in which the asymmetry of the dynamics occurs due to the change of the plant-transfer-function order. As the parameters of the plant increase, the dynamics of plant are described by the first-order transfer function, and as it decreases by the second-order transfer function. Cases when only the coefficients of the transfer function change, and the function order remains the same, are considered in the references [2,4]. In the literature [4], the modeling problem of a plant with asymmetric dynamics caused by a change in the coefficients of the transfer function is solved. The control problem of plants whose dynamic asymmetries are caused by the plant’s actuator-rate saturation change is analyzed in [5]. From the literature, Ref. [3] studies the control problem of the water level in a tank, and shows that such a plant has asymmetric dynamics. In analyzed references, the use two sets of controller parameter values for the control of plants with asymmetric dynamics is proposed. One set is used when the plant parameter increases, the other when the parameter decreases. 

Many real plants that have to be controlled are characterized by the response delay [6,7,8,9,10,11]. This happens with plants where the transport of some materials from a drive location to a specified location has to be provided. The plant’s response delay, if it is different to the rising-and-falling plant-control signal, also introduces the asymmetry of the plant dynamics. There are practically no publications devoted to plants with asymmetric dynamics caused by the asymmetry of the response delay. This problem is considered only in article [12], in which it the use the PI controller with switched parameters for the control of such plants is proposed. 

**Figure 1:**
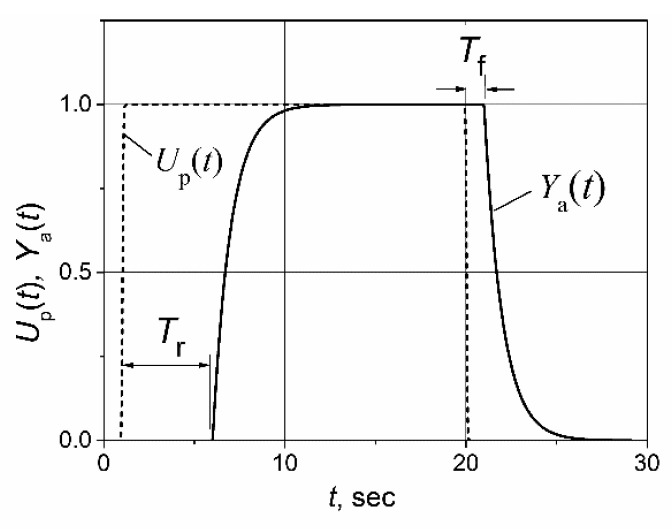
Unit pulse-response of the first-order plant *G*_p_(s) = 1/(1 + s)) with the asymmetric response delay to the rising-and-falling plant-control signal *U*_p_(*t*). *T*_r_ = 5 s, *T*_f_ = 1 s. *Y*_a_(*t*) is the plant’s output parameter.

The example of a unit’s pulse-response of a plant with an asymmetric response delay is shown in Figure 1. The transfer function of such a plant, including the delay term, is presented as follows:

$$G\left(s\right)=G_{p}\left(s\right)\;e^{-T_{p}\;s}\;,$$

(1) $$T_{p}=T_{r},\;\frac{dU_{p}}{dt}\geq 0,$$

$$T_{p}=T_{f},\;\frac{dU_{p}}{dt}<0\;,$$

where *G*_p_(s) is the plant-transfer function and *T*_r_ and *T*_f_ are the plant response-delays for the rising-and-falling plant-control signal *U*_p_ (*t*).

PI and PID controllers with a Smith predictor are often used to improve the control quality of plants with a response delay. There are many recent publications devoted to such controllers, e.g., [13,14,15,16,17,18,19]. However, there are no publications dealing with the application of the Smith predictor for control problems of plants with an asymmetric response delay.

The modification of a Smith predictor for the control of plants with response delay asymmetry has been proposed in this work. It was developed for application in frequency converters for the control of the speeds of the AC induction motor drives of pumps used in the water- and liquefied-petroleum-gas-supply systems. Such plants are characterized by a long response delay, and often this delay is asymmetric, i.e., the response delay to the rising-and-falling plant-control signal is different. A distinctive feature of the proposed modification is that the response delay in the model of the plant used for the realization of the Smith predictor is switched at the moment when the time derivative of the plant-control signal changes the sign. The operation of the proposed switched-delay Smith predictor, which was used with the proportional-integral controller, was investigated using a simulation and experimentally in the real domestic-water-supply system. The obtained results prove an advantage of the suggested solution.

All investigation results presented in Section 1, Section 2, Section 3 and Section 4 were obtained by modeling using Matlab/Simulink software, while the results presented in Section 5 were obtained by experimentally investigating a particular domestic-water-supply control system based on a variable-speed AC induction motor drive implemented using a frequency converter.

## 2. Problem Formulation

**Figure 2:**
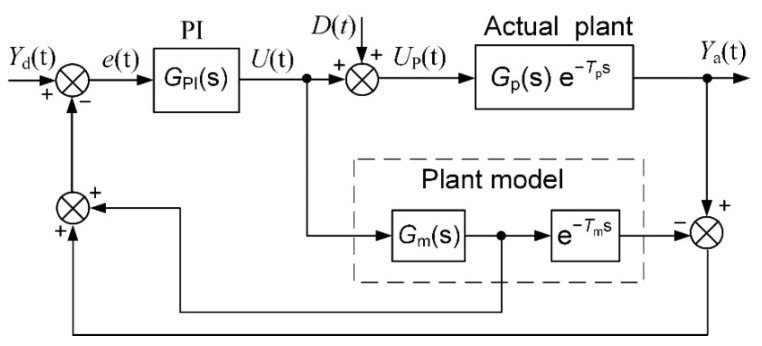
The block diagram of the control system based on the PI controller with the Smith predictor.

The principle of a Smith predictor for feedback-control systems with a response delay was proposed for the first time in [20]. The block diagram of the feedback-control system with the Smith predictor based on the PI controller is presented in Figure 2. The *Y*_d_(*t*) is the desired (set point) and *Y*_a_(*t*) is the actual value of the plant parameter, *e*(*t*) is the control error, *U*(*t*) is the controller’s output signal, *D*(*t*) is the load disturbance and *U*_p_(*t*) is the plant-control signal. *G*_PI_(s) is the transfer function of the PI controller, *G*_p_(s) is the plant-transfer function and e^−*T*p s^ is the plant-response delay term. *G*_m_(s) and e^−*Tm* s^ are the transfer function and the delay term of the plant model used for the implementation of the Smith predictor, respectively. 

The transfer function of the control system presented in Figure 2 is as follows [20]:

(2) $$\frac{Y_{a}\left(s\right)}{Y_{d}\left(s\right)}=\frac{G_{\mathrm{PI}}\left(s\right)\;G_{p}\left(s\right)\;e^{-T_{p\;}s}}{1+G_{\mathrm{PI}}\left(s\right)\;G_{p}\left(s\right)+G_{\mathrm{PI}}\left(s\right)\;G_{p}\left(s\right)\;e^{-T_{p\;}s}-G_{\mathrm{PI}}\left(s\right)\;G_{m}\left(s\right)\;e^{-T_{m}\;s}\;}$$

The plant model has to meet the condition *G*_m_(s) = *G*_p_(*s*) and *T*_m_ = *T*_p_ for the proper operation of the control system based on the Smith predictor [20]. The transfer function of the control system simplifies as follows in such a case:

(3) $$\frac{Y_{a}\left(s\right)}{Y_{d}\left(s\right)}=\frac{G_{\mathrm{PI}}\left(s\right)\;G_{p}\left(s\right)}{1+G_{\mathrm{PI}}\left(s\right)\;G_{p}\left(s\right)}\;e^{-T_{p\;}s},$$

**Figure 3:**
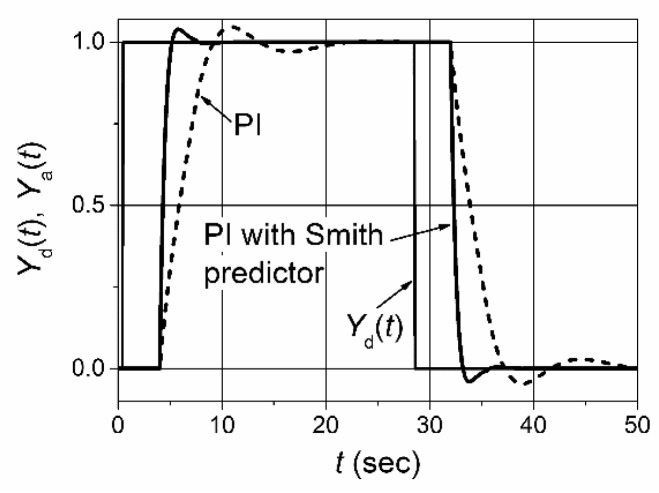
The set-point pulse-response of the control system based on the PI controller and the PI controller with the Smith predictor. The plant-transfer function including the delay term is *G*_p_(s) e^−*Tp* s^ = [1/(s + 1)] e^−3.5 s^. The parameters of the PI controller are *K*_p_ = 0.37, *K*_i_ = 0.18; the parameters of the PI controller used with Smith predictor are *K*_p_ = 2, *K*_i_ = 3; the transfer-function of the plant model in the Smith predictor is *G*_m_(s)= 1/(s + 1); the transport delay used in the model is *T*_m_ = 3.5 s.

The transfer function (3) demonstrates that the delay term is excluded from the feedback system and exists as separate external block, which simply adds the delay *T*_p_ to the response of the plant-output parameter. This fact allows for the controller to be tuned more aggressively. Therefore, the employment of the Smith predictor for the feedback-control of the plants with a response delay allows for the decrease of the main dynamic parameter—the settling time of the control system. This fact can be demonstrated by the pulse-response of the feedback-control system of the first-order plant with a response delay (Figure 3). It is seen that the implementation of the Smith predictor allows for the reducing of the settling time significantly. This is because the employment of the Smith predictor allows for the use of the PI controller with significantly higher values of parameters *K*_p_ and *K*_i_ without worsening the plant-response quality. This can be created because the delay term of the plant is removed from the feedback circuit when using the predictor and the controller can be tuned, as in the case of a plant without the delay. 

**Figure 4:**
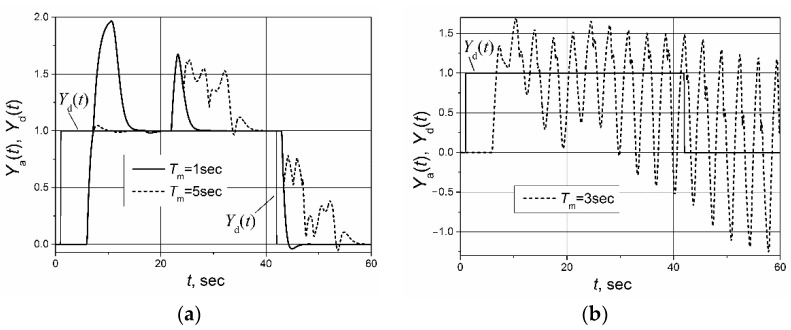
The set-point unit-pulse and unit-load disturbance-response of the control system of the first-order plant with the transfer function *G*_p_(s) = 1/(s + 1) and asymmetric response delay (*T*_r_ = 5 s, *T*_f_ = 1 s) based on the PI controller with the classical Smith predictor when (**a**) the delay of the model is adjusted for the values *T*_m_ = *T*_r_ = 5 s and *T*_m_ = *T*_f_ = 1 s, (**b**) the delay of the model adjusted for the values *T*_m_ = (*T*_r_ + *T*_f_)/2 = 3 s. Unit-load disturbance occurs at the time moment *t* = 17 s.

**Figure 5:**
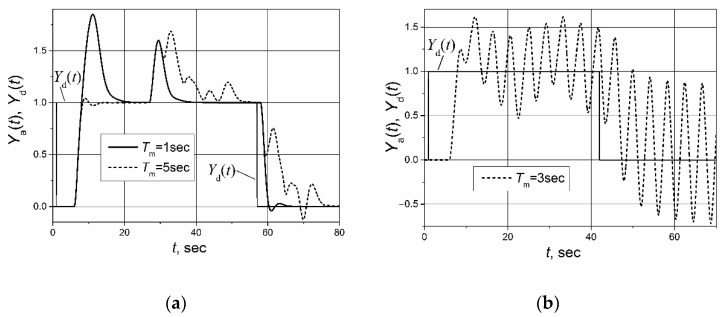
The set-point unit pulse and unit-load disturbance-response of the control system of the second-order plant with the transfer function *G*_p_(s) = 1/(s + 1)^2^ and asymmetric response delays (*T*_r_ = 5 s, *T*_f_ = 1 s) based on the PI controller with the classical Smith predictor when (**a**) the delay of the model is adjusted for the values *T*_m_ = *T*_r_ = 5 s and *T*_m_ = *T*_f_ = 1 s, (**b**) the delay of the model is adjusted for the value *T*_m_ = (*T*_r_ + *T*_f_)/2 = 3 s. The unit-load disturbance occurs at the time moment *t* = 22 s.

The control system with the implemented Smith predictor operates properly when the transfer function and response delay of the plant model match the plant-transfer function and -response delay [21,22,23,24,25]. Therefore, application of the classical Smith predictor (Figure 2) for the control of plants with response-delay asymmetry is complicated. To demonstrate this, the simulation of the set-point unit-pulse and unit-load-disturbance response of the control system based on the PI controller with the classical Smith predictor of the plants described by the first- and second-order transfer functions with the asymmetric response delay was performed. Such plants were chosen for analysis because the dynamics of many real controlled plants can be described by the first- or second-order transfer function plus the response delay [26,27,28,29,30]. The obtained results are presented in Figure 4 and Figure 5. It is seen that if the response delay in the plant model *T*_m_ is adjusted for the delay of the plant’s response to the rising plant-control signal, the plant’s response to the falling plant-control signal and its response to the load disturbance are characterized by a long settling time (Figure 4a and Figure 5a dashed lines). In the case when the response delay in the plant model is adjusted for the plant-response delay to the falling plant-control signal, the transient of the plant response to the rising plant-control signal has a huge overshoot (Figure 4a and Figure 5a, solid lines).

If the plant-model delay time is adjusted for the average of the response delays to the rising-and-falling plant-control signals *T*_m_ = (*T*_r_ + *T*_f_)/2, the control system operates not stably (Figure 4b and Figure 5b). The responses presented in Figure 4 and Figure 5 and the responses presented in other sections of this article were obtained for following parameters of the PI controller used with the Smith predictor: for the control systems with the first-order plant, *K*_p_ = 1.7, *K*_i_ = 2.7; for the control systems with the second-order plant, *K*_p_ = 1.59, *K*_i_ = 0.88. 

## 3. Switched-Delay Smith Predictor

**Figure 6:**
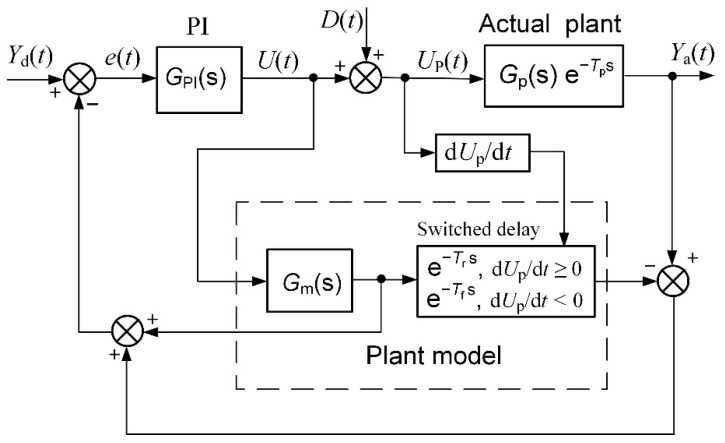
The block diagram of the control system based on the PI controller with the switched-delay Smith predictor.

The results of this investigation of the control systems of first- and second-order plants with asymmetric response delay based on the PI controller with the classical Smith predictor (Figure 4a and Figure 5a) lead to a conclusion that for the proper operation of the control system of a plant with response-delay asymmetry, the response delay *T*_m_ in a model of the plant has to be switched. The block diagram of a feedback-control system based on the PI controller with the proposed switched-delay Smith predictor is presented in Figure 6. The delay is switched at the moment when the time derivative of the plant-control signal d*U*_p_/d*t* changes the sign. Compared with the classical Smith predictor, the switched-delay Smith predictor has two new functional blocks: one block that observes the sign of the plant-control signal-time derivative d*U*_p_/d*t* and one switched-delay block which sets the value of the response delay *T*_m_ in the plant model dependent on the derivative d*U*_p_/d*t* sign.

**Figure 7:**
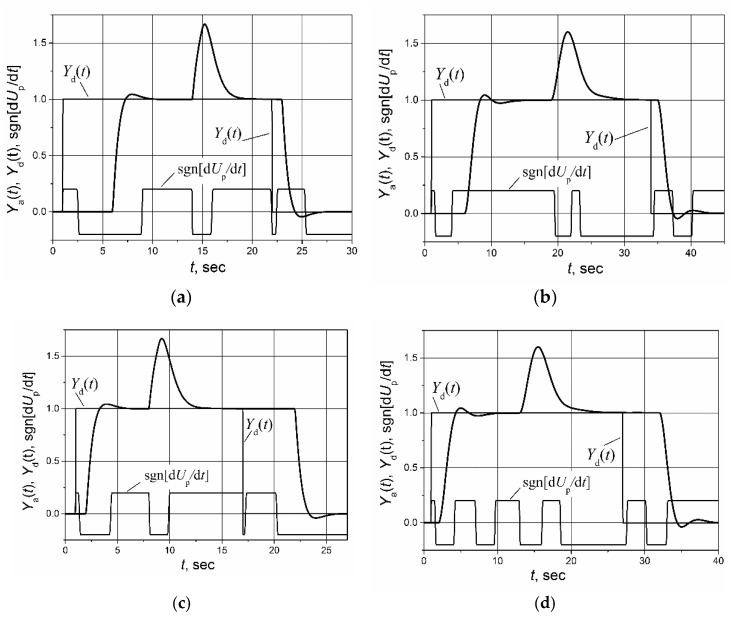
The set-point unit pulse and unit-load disturbance-response of the control system of the first-order (**a**) and second-order (**b**) plants with asymmetric response delay (*T*_r_ = 5 s, *T*_f_ = 1 s) and the first-order (**c**) and second-order (**d**) plants with asymmetric response delay (*T*_r_ = 1 s, *T*_f_ = 5 s) based on the PI controller with the switched-delay Smith predictor. Load disturbance occurs at the time moments *t* = 9 s (**a**), *t* = 14 s (**b**), *t* = 7 s (**c**) and *t* = 12 s (**d**). The sgn[d*U*_p_/d*t*] is the sign of the plant-control signal time derivative.

For the evaluation of the PI controller with the switched-delay Smith predictor, the set-point unit pulse and unit-load disturbance-response of the control system of the first-order plant with the transfer function *G*_p_(s) = 1/(s + 1) and the second-order plant with the transfer function *G*_p_(s) = 1/(s + 1)^2^ with the asymmetric response delays *T*_r_ = 5 s, *T*_f_ = 1 s and *T*_r_ = 1 s, *T*_f_ = 5 s were simulated. The results are presented in Figure 7. The sgn[d*U_p_*/d*t*] in Figure 7 provides the information about the sign of the plant-control signal-time derivative. It is seen that the proposed modification allows for the maintaining of the capabilities of the Smith predictor in the case of asymmetric dynamics, i.e., the response of the control system with the switched-delay Smith predictor to both rising and falling control signals is characterized by a short settling time and low overshoot.

A specific feature of the proposed Smith predictor modification is that it is necessary to observe the change of the sign of the plant-control signal-time derivative d*U*_p_/d*t* and, accordingly, switch the response-delay time in the plant model. It is not problematic to measure the sign of the derivative d*U*_p_/d*t* when analyzing the operation of the control system using a simulation because the disturbance signal *D*(t) in the simulation model is simply added to the controller-output signal *U*(*t*) that is fed to the control input of the object, i.e., *U*_p_(*t*) = *U*(*t*) + *D*(t) (Figure 6). Meanwhile, in practice, load disturbance affects in other ways, for example in the water-supply system, when closing or opening the water valve, and it does not add up directly to the controller-output signal. Of course, when a load disturbance occurs, the control error *e*(*t*) changes and, as a result, the controller-output signal changes as well and, consequently, the controller-output (plant-control input) signal-time derivative changes, and a block that observes the sign of d*U_p_*/d*t* will capture it. However, this would be fine for a plant with no response delay. If there is a delay, the response of the controller-output signal to the load disturbance will also be delayed, so the delay term in the plant model of the Smith predictor would be switched with a delay in response to the load disturbance. Therefore, to respond to a load disturbance without delay, an additional signal related to the load disturbance should be submitted to the switched-delay block of the Smith predictor. It is not necessary to obtain the accurate quantitative value of the disturbance effect. This signal just has to provide the information about the polarity of the disturbance.

In the literature [12] the asymmetric PI (aPI) controller for the controlling of plants with the asymmetric response delay has been proposed, so it is interesting to compare it with this one proposed in this paper based on the PI controller with the switched-delay Smith predictor. 

The control algorithm of an aPI controller is as in the following [12]:

(4) $$\begin{array}{l} U\left(t\right)=K_{p}(t)\;e\left(t\right)+\int_{t_{0}}^{t}K_{i}(\tau )\;e\left(\tau \right)\;d\tau ,\\ K_{p}\left(t\right)=K_{\mathrm{pp}},K_{i}(\tau )=K_{\mathrm{ip}}\;|\;e\left(t\right)\geq 0\;,\\ K_{p}\left(t\right)=K_{\mathrm{pn}},K_{i}(\tau )=K_{\mathrm{in}}\;|\;e\left(t\right)<0\;, \end{array}$$

where *U*(*t*) is the controller-output signal, *K*_pp_, *K*_ip_ and *K*_pn_, *K*_in_ are the proportional and integral constants used at the positive and at negative control errors *e(t)*, respectively, and *t_0_* is the moment the algorithm starts working.

It is necessary to note that the aPI controller not only has a different control algorithm compared with the one proposed in this article, but it also has a different method of determining the switching moments of the controller parameters. The parameters in the aPI controller are switched at the time when the control error *e*(*t*) changes its sign, while in the controller proposed in this article the value of the delay time of the plant model *T*_m_ is switched when the sign of the plant-control signal time derivative d*U*_p_/d*t* changes. Furthermore, in the proposed controller there is no need to switch the parameters of the PI controller.

**Figure 8:**
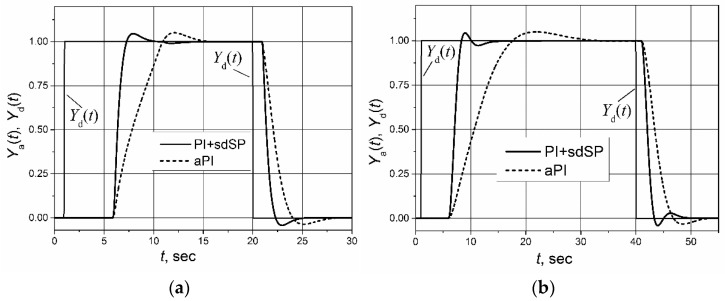
The set-point unit pulse response of the control system of the first-order (**a**) and second-order plants (**b**) with asymmetric response delay (*T*_r_ = 5 s, *T*_f_ = 1 s) based on the asymmetric PI (aPI) controller and the PI controller with the switched-delay Smith predictor (sdSP).

The comparison of the set-point unit pulse-response of the control system of the first-order and second-order plants with the asymmetric response delay based on the aPI and PI controllers with the switched-delay Smith predictor are given in Figure 8. The parameters of the aPI controller for the first-order plant with an asymmetric response delay are *K*_pp_ = 0.42, *K*_ip_ = 0.15, *K*_pn_ = 0.46 and *K*_in_ = 0.48 and for the second-order plant are *K*_pp_ = 0.23, *K*_ip_ = 0.105 and *K*_pn_ = 0.4, *K*_in_ = 0.29. It is seen that application of the switched-delay Smith predictor allows for the provision of a shorter settling time of the plant response as compared with the case when an aPI controller is used. A shorter settling time is achieved because of the application of the Smith predictor. It eliminates the plant-response delay from the feedback circuit (see Formula (3)); therefore, the values of the controller parameters *K*_p_ and *K*_i_ can be the same as for the control of the plant without a response delay, i.e., they can be increased without causing a large overshoot of the plant response.

## 4. Analysis of Robustness

The dynamic parameters of real controlled plants can change as their working conditions change. The robustness of the control system characterizes the ability of the system to maintain stable operations in cases when plant parameters vary; therefore, it is a particularly important characteristic in the practical applications of control systems. The operation of control systems based on controllers with the Smith predictor are sensitive to changes in the response delay of the controlled plant [25,31]. Therefore, it is of interest to investigate how the analyzed control systems based on the switched-delay Smith predictor withstand variations in the response delay of the plant with the asymmetric dynamics. The analysis was performed for the plants with the first and second order transfer functions *G*_p_(s) = 1/(s + 1) and *G*_p_(s) = 1/(s + 1)^2^ with an asymmetric response delay (*T*_r_ = 5 s, *T*_f_ = 1 s). The investigation was made for the following situations: (a) only the response delay to the rising control signal of the plant changes; (b) only the response delay to the falling control signal of the plant changes; (c) at the same time, the response delay to both rising and falling control signals of plant changes. The maximum range of plant-response-delay variation was from −50% to +100%. In all cases, the delay in plant model of the switched-delay Smith predictor was adjusted for *T*_r_ = 5 s and *T*_f_ = 1 s. The PI controller parameters were the same as in the investigations described above.

**Figure 9:**
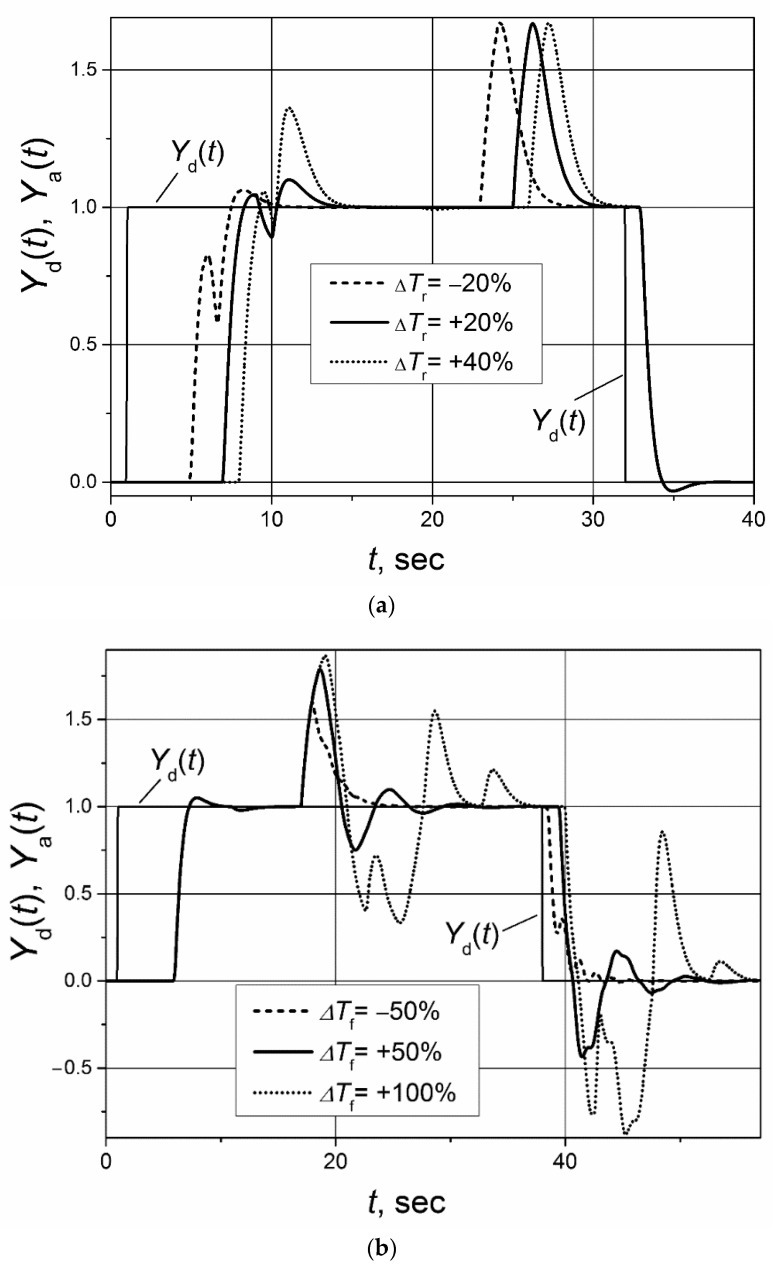
The set-point unit pulse and unit-load disturbance responses of the control system of the first-order plant with the asymmetric response delay based on the PI controller with the switched-delay Smith predictor when the plant-response delay changes: (**a**) changes just *T*_r_ by value ∆*T*_r_; (**b**) changes just *T*_f_ by value ∆*T*_f_. Unit-load disturbances occur at times: *t* = 19 s (**a**); t = 12 s (**b**).

**Figure 10:**
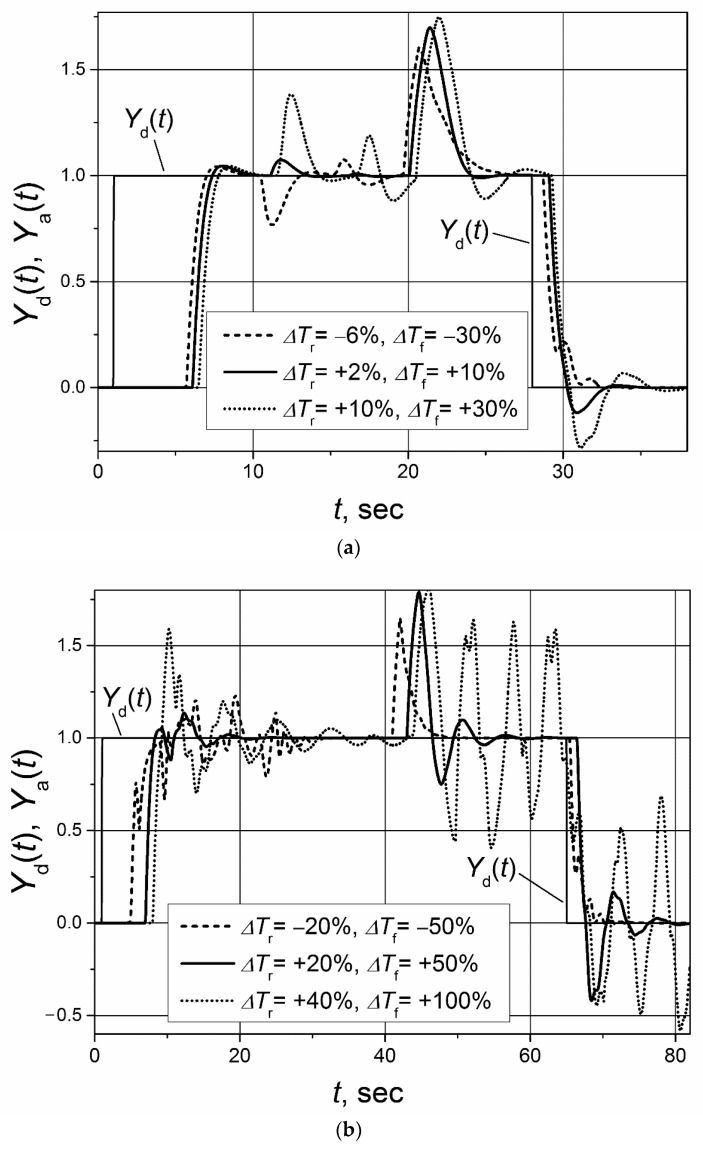
The set-point unit pulse and unit-load disturbance responses of the control system of the first-order plant with asymmetric response delay based on the PI controller with the switched-delay Smith predictor when both plant-response delays change: *T*_r_ changes by value ∆*T*_r_ and *T*_f_ by value ∆*T*_f_. Unit-load disturbances occur at times: *t* = 15 s (**a**); *t* = 36 s (**b**).

**Figure 11:**
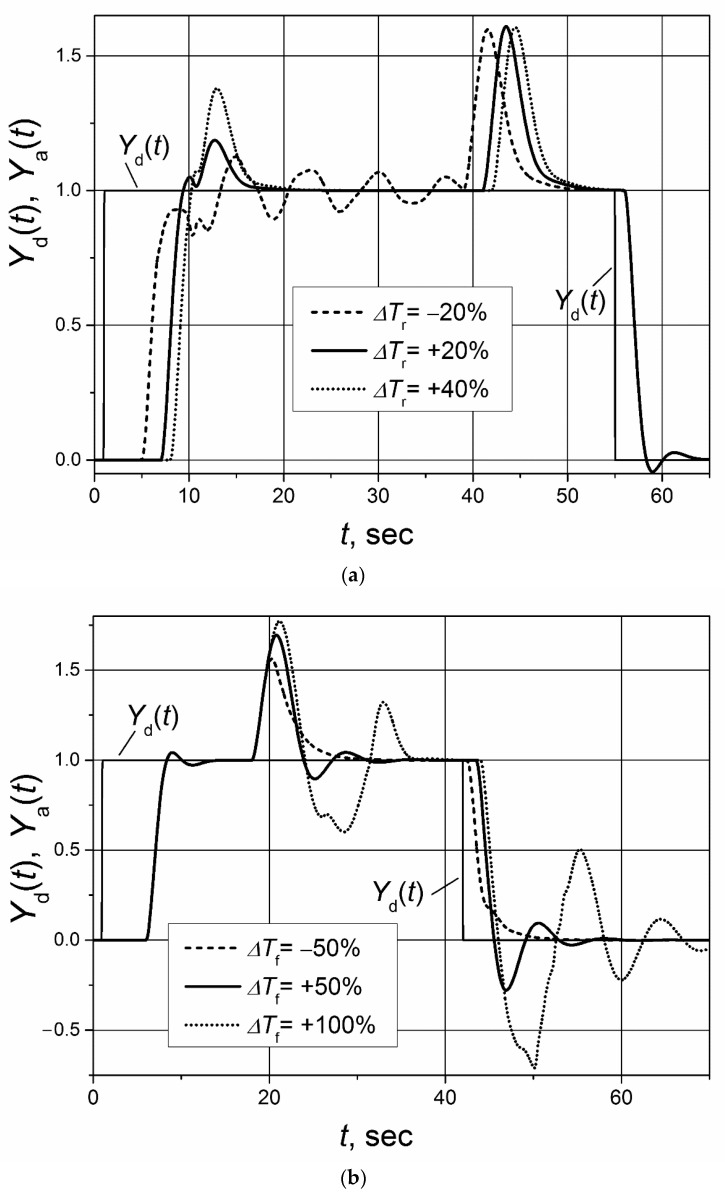
The set-point unit pulse and unit-load disturbance responses of the control system of the second-order plant with asymmetric response delay based on the PI controller with the switched-delay Smith predictor when plant-response delay changes: (**a**) changes just *T*_r_ by value ∆*T*_r_; (**b**) changes just *T*_f_ by value ∆*T*_f_. Unit-load disturbances occur at times: (**a**) *t* = 35 s; (**b**) *t* = 13 s.

**Figure 12:**
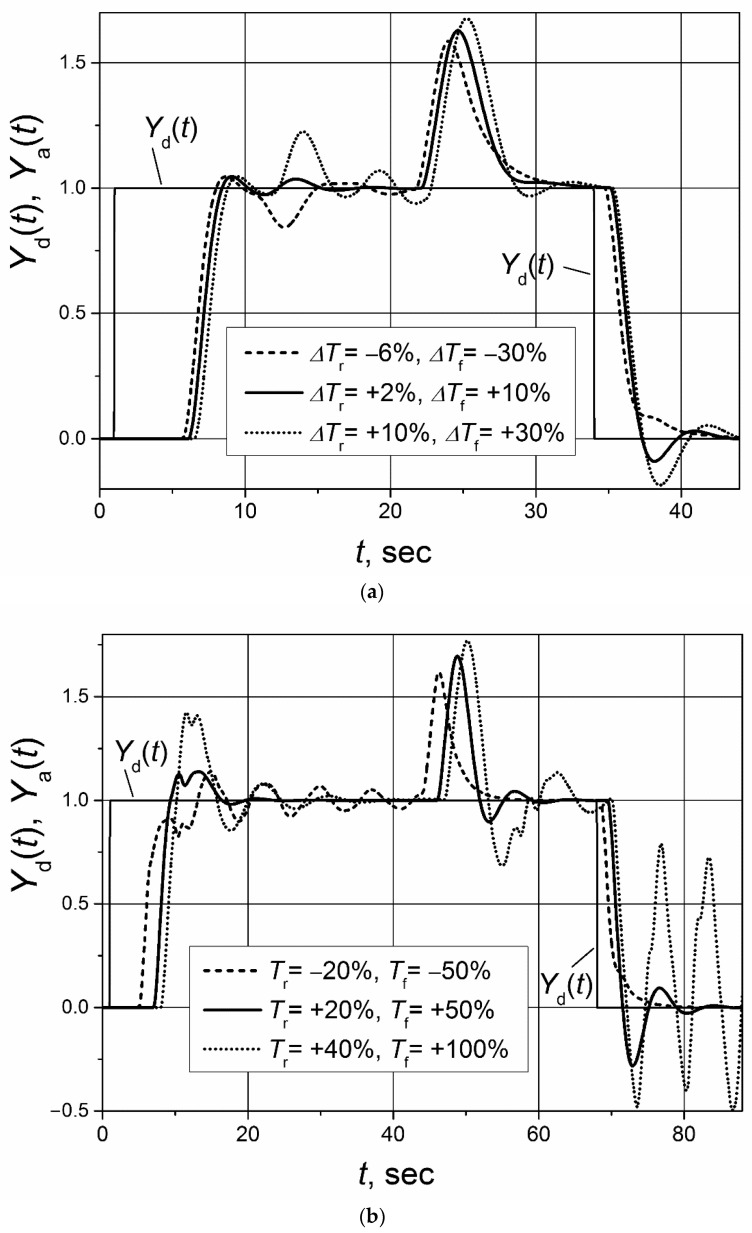
The set-point unit pulse and unit-load disturbance responses of the control system of the second-order plant with asymmetric response delay based on the PI controller with the switched-delay Smith predictor when both plant-response delays change: *T*_r_ changes by value ∆*T*_r_ and *T*_f_ by value ∆*T*_f_. Unit-load disturbances occur at times: (**a**) *t* = 17 s; (**b**) *t* = 40 s.

The obtained analysis results for the control system of the first-order plant are presented in the Figure 9 and Figure 10 and for the control system of the second-order plant in Figure 11 and Figure 12. They show that the change of the plant-response delay increases the settling time and overshoots of plant response and even that the system may start to operate in an unstable manner. When the plant’s response delay decreases, the increase of overshoots and pulsation amplitudes in the response transients is lower compared with the case when the response delay increases. Therefore, it can be concluded that the cases when the plant-response delay increases are more dangerous for the control system’s operation stability. It is seen that the analyzed control systems of the first- and second-order plants with the asymmetric response delay remain stable if an increase of plant-response delay to the rising and falling plant control signal ∆*T*_r_ ≤ 40% and ∆*T*_f_ ≤ 100%. If those limits are exceeded, the control system starts to work in an unstable manner (see Figure 10b and Figure 12b). 

## 5. Experimental Investigation

**Figure 13:**
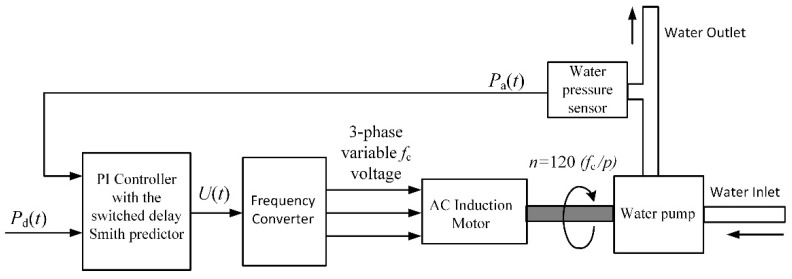
Block diagram of the water-supply control system based on the variable speed AC induction motor drive implemented using frequency converter. *P*_d_(*t*) is the desired and *P*_a_(*t*) is the actual water pressure, *f*_c_ is the frequency of 3-phase voltage generated by the frequency converter, *n* is the motor-rotation speed, *p* is the number of motor poles.

The proposed controller based on the switched-delay Smith predictor was investigated in a specialized frequency converter for controlling the speed of AC induction motors of water pump drives in domestic water-supply systems. The block diagram of such a water-supply system is presented in Figure 13. The purpose of the control system is to maintain the desired water pressure *P*_d_(*t*), regardless of water consumption.

**Figure 14:**
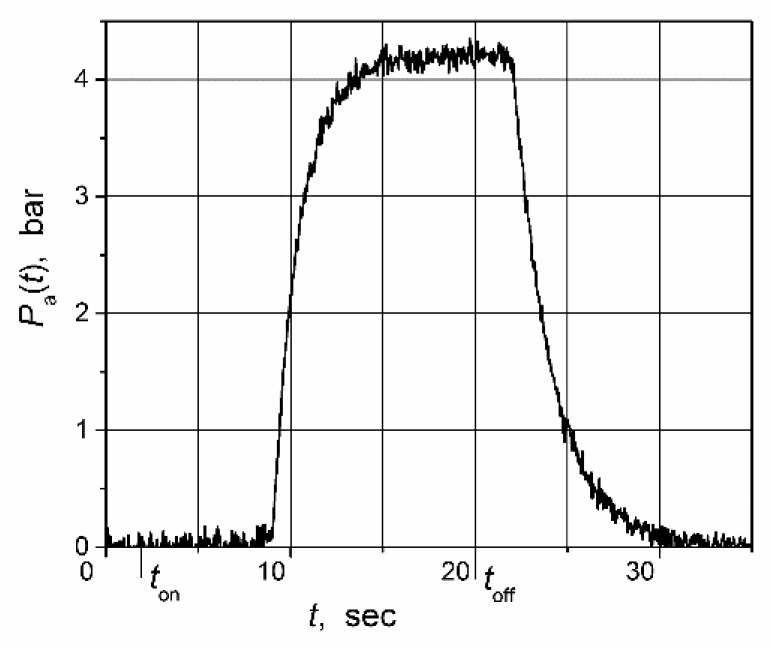
Open-loop pulse response of the water pressure of water supply system. The AC motor drive was switched on at time moment *t*_on_ = 2 s, and shut down at time moment *t*_off_ = 20 s.

The pulse-response of the water pressure in the water-supply system of a particular five-story apartment building where the control system was tested is presented in Figure 14. It is seen that the response delay is asymmetric, with *T*_r_ = 7 s and *T*_f_ = 2 s. Additionally, it can also be observed that the dynamics of the water-supply system during the rise and fall of water pressure is noticeably different. From the pulse response of the water-supply system, it can be determined that the dynamics of the investigated water-supply system can be presented by the first-order transfer function with the 1.4 s time constant when the pressure is rising and with the 2.0 s when it is falling. This fact supports the claim that the dynamics of real plants are often more-or-less asymmetric. The transfer function of the water-supply system model *G*_m_(s) = 1/(1.7 s + 1) was used for the implementations of the classical and switched-delay Smith predictors. The time constant (1.7 s.) used in this function is the average of the obtained time constants for the rising and falling of the water pressure. 

The water pressure set-point pulse and the load-disturbance response of the water-supply control system were investigated. The investigation was performed for the cases when the PI controller with the switched-delay Smith predictor and with the classical Smith predictor were used. The control system with the classical Smith predictor was investigated for the fallowing three cases of plant-model time-delay values: *T*_m_ = *T*_f_ = 2 s; *T*_m_ = (*T*_r_ + *T*_f_)/2 = 4.5 s; *T*_m_ = *T*_r_ = 7 s. The investigation was conducted in the following manner: the set point (desired value) of the water pressure *P*_d_(*t*) was set to four bars and the water-control system was switched on; 33 s after switching on, a load disturbance was made for the system, closing one of the water outlet valves through which the water was consumed; 66 s after switching on, the desired water pressure was set to 1.6 bar value. We used an additional sensor to fixate the closing moment of the water valve. The signal of the sensor was used for the determination the switching moment for the delay term of the plant model in the switched-delay Smith predictor, responding to the load disturbance.

**Figure 15:**
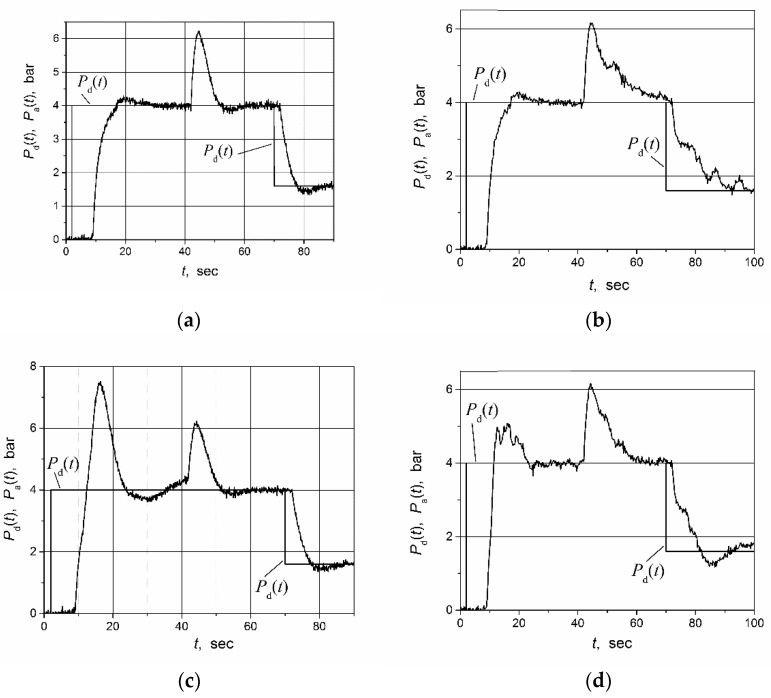
The water pressure set-point *P*_d_(*t*) pulse (set point 4 bar step followed by drop up to 1.6 bar) and load-disturbance responses of the water-supply control system. The system is based on the PI controller with the switched-delay Smith predictor (**a**) and the classical Smith predictor (**b**–**d**). The delays in the model of the switched-delay Smith predictor were adjusted to values *T*_r_ = 7 s and *T*_f_ = 2 s, and in the classical Smith predictor to values (**b**) *T*_m_ = 7 s; (**c**) *T*_m_ = 2 s; (**d**) *T*_m_ = 4.5 s. The PI controller parameters in both control systems were tuned to values *K*_p_ = 0.85 and *K*_i_ = 0.57, the transfer function of the water-supply system model used in both controllers was *G*_m_(s) = 1/(1.7 s + 1).

The obtained results of experimental investigation are presented in Figure 15.

The experimental investigation’s results support the results obtained through the simulation. We can see (Figure 15a) that applying the PI controller with the switched-delay Smith predictor allows for us to obtain a good transient quality with the short settling time and low overshoot during the rising and falling of the water pressure. However, the application of the PI controller with the classical Smith predictor does not allow for us to obtain a good transient quality for the control of the analyzed water-supply control system. When the time delay in the model of the plant is adjusted for the response delay to the rising control signal of the plant (*T*_m_ = *T*_r_), pulsations appear in the transient during the falling of water pressure, and the settling time significantly increases (see Figure 15b) as compared with the situation when the switched-delay Smith predictor is employed. When the time delay in the model of the water-supply system is adjusted for the response to the falling control signal of the plant (*T*_m_ = *T*_f_), a huge overshoot is observed during the rising of the water pressure (Figure 15c). If the model’s time delay is adjusted for the average delay value *T*_m_ = (*T*_r_ + *T*_f_)/2 = 4.5 s, the transient is proper neither during the rising nor during the falling of the water pressure because it is characterized by a long settling time and a high overshoot (Figure 15d). It can also be seen that the response to the load disturbance of the water-supply control system based on the classical Smith predictor has a longer settling time, as in the case when the switched-delay Smith predictor is used (compare transients presented in Figure 15b,d with the one given in Figure 15a). 

The high-frequency pulsations seen in the transients presented in Figure 14 and Figure 15 are caused by the electromagnetic disturbances produced by the output stage of the frequency converter.

## 6. Discussion

Many real plants have asymmetric dynamics, however, there is relatively low number of works dedicated to the feedback-control problems of such plants. In most cases, feedback-control problems are solved assuming that the plant has symmetric dynamics. Due to this, usually, the step response, but not the pulse response, has been analyzed when examining the control system in the time domain. However, in the case of high asymmetry it has to be taken into consideration, and in order to obtain a suitable control quality, it is necessary to modify the existing control methods by adapting them to the control of plants with asymmetric dynamics. 

The problem addressed in this work arose when applying frequency converters for the speed control of water and liquefied-petroleum gas-pump drives based on the AC induction motors. Due to the fact that such systems are used to transport materials from one point to another, they are characterized by long response delays. Therefore, it was suggested to use a Smith-predictor-based controller to obtain the proper dynamics of the control system. Due to the fact that the response delays to the rising and falling control signals of the water- and liquefied-petroleum-gas-supply systems were different, it was necessary to modify the Smith predictor by adapting it to such specifics of the plants.

The proposed switched-delay Smith predictor uses two delay values in the plant model, one for the response to the rising plant-control signal, the other to the falling plant-control signal. This improvement allows for the proper control-system response transient with the short settling time and low overshoot to be obtained during the rise and fall of the plant output when controlling plants with the response-delay asymmetry. An analysis of the control robustness of the first- and second-order plants with the response-delay asymmetry, when the delay to the rising control signal is 5 s, to a falling 1 s using the PI controller with the switched-delay Smith predictor, shows that the control system works stably if the increases of the plant’s response delay to the rising and falling plant-control signal are less than 40% and 100%, respectively. When the plant’s response delay decreases, the increase of overshoots and pulsation amplitudes in the response transients is lower compared with the case when the response delay increases. Therefore, it can be concluded that the cases when the plant’s response time increases are more dangerous for the control system’s operation stability.

The experimental investigation of the proposed switched-delay Smith predictor for the control of water pressure in the water-supply system of a particular five-story apartment building using a frequency converter for controlling the speed of the AC induction motor of a water pump drive supports the results obtained through the simulation and proves an advantage of the suggested solution.

Due to the fact that asymmetric dynamics introduced by the plant’s response-delay asymmetry are often encountered in the real applications of control systems, it is likely that the investigation’s results presented in the paper could be useful to a wider audience facing similar problems in the applications of feedback-control systems.

## Data Availability

Not applicable.
